# Supplementary material for: Susceptibility of Fat Tissue to SARS-CoV-2 Infection in Female hACE2 Mouse Model
Source: Int J Mol Sci. 2023 Jan 9;24(2):1314. doi: 10.3390/ijms24021314 (PMC9863100; doi:10.3390/ijms24021314)
Supplement: Supplementary file 1 [file ijms-24-01314-s001.zip › ijms-2000568-supplementary.docx]

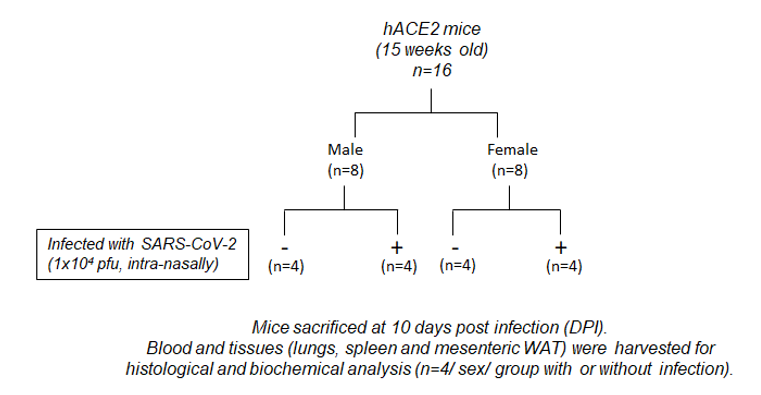


**Figure S1.** Flow chart of the experimental design.


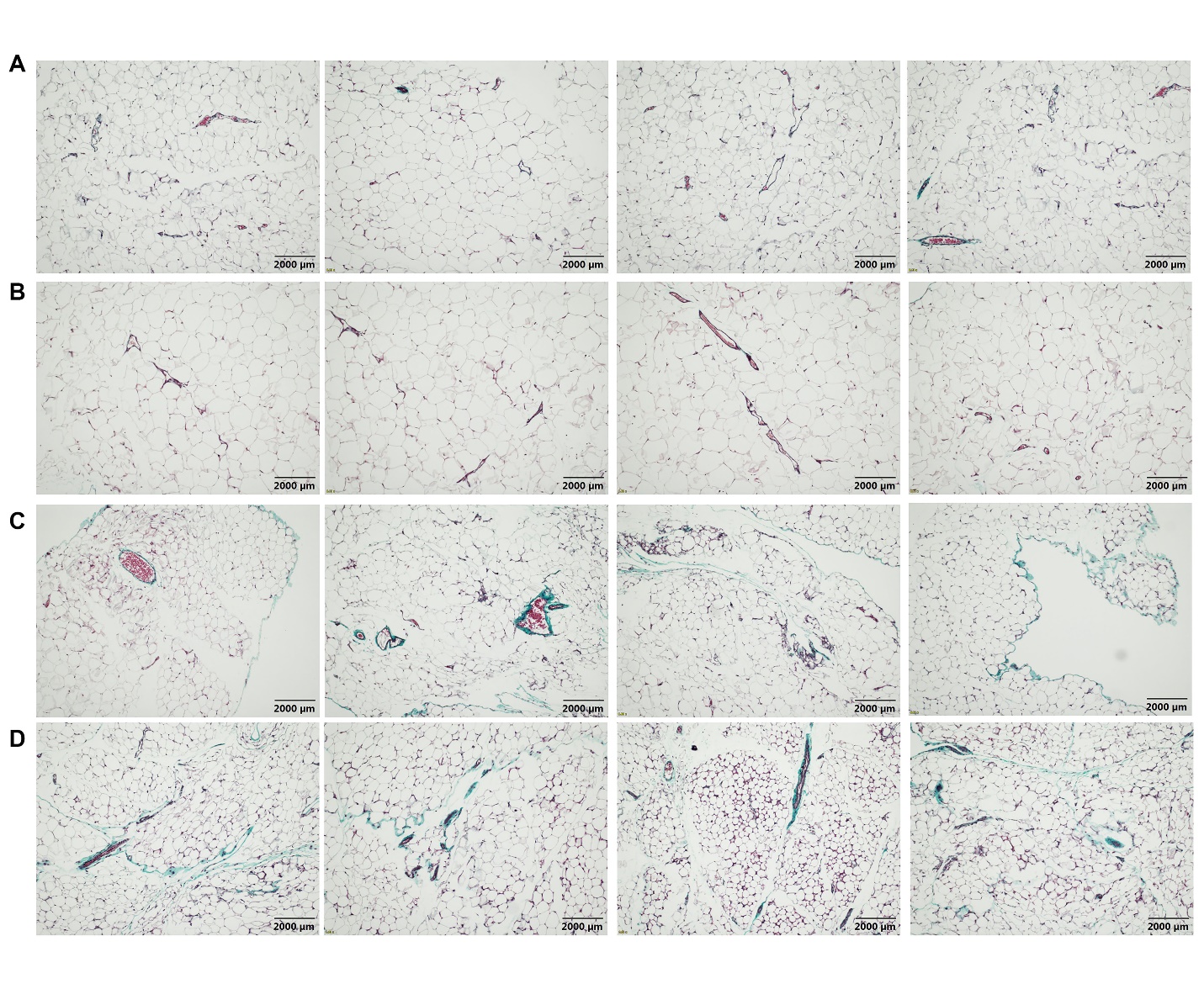
**Figure S2.** Masson-trichrome staining of WAT sections showing fibrosis in CoV-2 infected hACE2 mice (n=4 mice/sex). (**A**) Uninfected male; (**B**) Uninfected female; (**C**) CoV-2 infected male; and (**D**) CoV-2 infected female (10x magnification, scale bar - 200 µm).
